# Identification and verification of the key genes, CCR1 and EGR2, in diabetes-associated lipophagy

**DOI:** 10.1038/s41598-026-43737-9

**Published:** 2026-03-20

**Authors:** Jiongjiong Liu, Xiao Zhang, Yanlei Wang, Wenjun Pei, Qiu Zhang

**Affiliations:** 1 Department of Endocrinology, The First Affiliated Hospital of Anhui Medical University, Hefei, 230022 Anhui People’s Republic of China; 2 Department of International Medical Service, The First Affiliated Hospital of USTC, Division of Life Sciences and Medicine, University of Science and Technology of China, Hefei, 230001 Anhui People’s Republic of China; 3 Department of Paediatric Surgery, The First Affiliated Hospital of USTC, Division of Life Sciences and Medicine, University of Science and Technology of China, Hefei, 230001 Anhui People’s Republic of China; 4 Anhui Province Key Laboratory of Basic Research and Transformation of Age-related Diseases, Wannan Medical College, Wuhu, 241002 People’s Republic of China

**Keywords:** Diabetes, Lipophagy, CCR1, EGR2, Biomarkers, Computational biology and bioinformatics, Diseases, Endocrinology

## Abstract

Diabetes remains a significant global health challenge, marked by increasing incidence and a complex pathophysiological mechanism involving dysregulated lipid metabolism, impaired autophagy, and chronic inflammatory responses. Lipophagy, an autophagic process that involves the targeting of lipid droplets, is crucial for metabolic homeostasis. Therefore, investigating lipophagy-associated molecules may facilitate the discovery of novel biomarkers and potential therapeutic targets for diabetes. In this study, two GEO datasets, GSE33440 and GSE9006, were combined to identify differentially expressed genes (DEGs) linked to diabetes. By integrating weighted gene coexpression network analysis (WGCNA) with machine learning algorithms, this study identified *EGR2* and *CCR1* as key hub genes related to lipophagy. The results of the rank sum test revealed a strong positive correlation between these two genes, both of which were significantly upregulated in diabetic samples. Functional analyses, such as gene set enrichment analysis (GSEA), gene ontology (GO) enrichment, and protein‒protein interaction (PPI) network analysis, were used to validate their coherence. Diagnostic models and receiver operating characteristic (ROC) curve analysis further underscore the potential of *CCR1* and *EGR2* as biomarkers. Importantly, experimental validation demonstrated that the expressions of both genes were significantly elevated in the serum of diabetic patients and in the liver tissues of BKS-db diabetic mice. Notably, compared with control mice, *CCR1* knockout mice (*CCR1*-/-) exhibited improved glucose homeostasis under high-fat diet conditions. Collectively, these findings suggest that *EGR2* and *CCR1* may be potential biomarkers associated with lipophagy in diabetes.

**Supplementary Information:**

The online version contains supplementary material available at 10.1038/s41598-026-43737-9.

## Introduction

Diabetes is a chronic metabolic disorder that is characterized by insulin resistance and relative deficiency^1^. Patients with type 2 diabetes mellitus (T2DM) frequently exhibit characteristics of metabolic syndrome, including obesity, hypertension, and hyperlipidaemia, which collectively increase the risk of heart disease^2^. However, the incidence of diabetes has greatly increased over the past few decades, becoming one of the major global health challenges. According to the International Diabetes Federation (IDF), approximately 537 million adults globally had diabetes in 2021, with the majority having T2DM^3^. Despite the availability of various treatment options, the complicated nature of the disease and the large differences between individuals often result in unsatisfactory treatment outcomes.

The pathogenesis of T2DM involves multiple factors, but discussions regarding lipid metabolism issues are ongoing. Research has indicated that lipid metabolism disorders are strongly linked to the onset of T2DM, particularly insulin resistance caused by excessive lipid accumulation. The accumulation of natural lipids can inhibit the phosphorylation of insulin receptor substrates (IRSs), disrupting the normal function of insulin^4^. In T2DM patients, excessive lipid accumulation is closely associated with metabolic disorders in various organs, including fat deposition in the liver and muscles. Nonalcoholic fatty liver disease (NAFLD) is directly associated with excessive lipid accumulation^5^. Furthermore, the accumulation of lipid droplets in muscles can lead to decreased insulin sensitivity^6^. Notably, excessive lipid accumulation can initiate the release of inflammatory substances in the body, leading to a series of metabolic disorders and tissue damage. Tumour necrosis factor-alpha (TNF-α) and interleukin-6 (IL-6) contribute to the development of insulin resistance^7,8^. These results emphasize the close, bidirectional and causally interrelated connection between lipid metabolism and blood glucose.

Autophagy is a self-degradation process in cells that can result in the removal of misfolded proteins and damaged organelles. It can also eliminate intracellular pathogens and is generally considered a survival mechanism^9^. Research has indicated that autophagy genes play a role in diverse phenotypes and human diseases, such as neurodegenerative^10^, liver^11^, muscle^12^, cancer^13^, and cardiovascular diseases^14^. In diabetes, the process of autophagy is dysregulated^15^. The positive effects of autophagy include enhancing β-cell function^16^, improving muscle atrophy^17^ and delaying cognitive impairment^18^. Lipophagy, a type of autophagy, is essential for the regulation of lipid metabolism^19^. However, this specific autophagy mechanism has not been fully studied, especially the genes linked to lipophagy in diabetes, which remain largely unknown and require further exploration.

Although existing studies have recognized the roles of lipid metabolism disorders and autophagic dysregulation in diabetes progression, the understanding of lipophagy—the crucial link between them—remains inadequate given its regulatory mechanisms and clinical translation potential in diabetes. There is currently a lack of interdisciplinary research that integrates genomic data with experimental validation, which hinders the translation of the lipophagy theory into clinical applications.

Therefore, this study employs a combined approach involving bioinformatics and experimental validation to systematically analyse the function of lipophagy in the pathophysiology of diabetes. Its objective is to leverage machine learning approaches for more accurate identification of key gene networks and screening of biomarkers with diagnostic or therapeutic potential^20^. This research not only seeks to elucidate the molecular mechanisms of the “lipophagy–diabetes” regulatory axis but also provides a foundation for its clinical translation, facilitating the development of new diagnostic and therapeutic strategies.

## Methods

### Data acquisition

This study utilized publicly accessible data from the Gene Expression Omnibus website (GEO, https://www.ncbi.nlm.nih.gov/geo/). Diabetes-related genome-wide expression profiles were obtained from the GEO via the R package “GEOquery”. Dataset GSE33440 (GPL6947 Illumina HumanHT-12 V3.0 expression beadchip) included 16 whole blood samples from diabetes patients and 6 from healthy controls, whereas GSE9006 (GPL96 [HG-U133A] Affymetrix Human Genome U133A Array and GPL97 [HG-U133B] Affymetrix Human Genome U133B Array) included 93 peripheral blood mononuclear cell (PBMC) samples from diabetes patients and 24 from healthy controls. Batch effects caused by nonbiological technical variations were corrected using the ComBat method from the R package “sva”^21^. The effectiveness of the corrections was assessed via principal component analysis (PCA). The PCA results indicated that no obvious batch effects remained among the processed samples (Fig. S1A-B). The study adhered to the data access policies of all the participating databases. A total of 803 autophagy-related genes (Table S1) were compiled from the Human Autophagy Moderator database (HAMdb) and the Human Autophagy Database (HADb). A total of 1,564 genes associated with lipid metabolism were sourced from the MSigDB database (https://www.gsea-msigdb.org/gsea/msigdb/), as detailed in Table S2.

### Diabetes-related differential analysis

The R package “limma” (version 3.50.0)^22^ was used to detect differentially expressed genes (DEGs) between the control group (*n* = 30) and the diabetes group (*n* = 109). Differentially expressed genes (DEGs) were identified using screening criteria of |log2Fold Change| > 0.5 and *P* < 0.05 for further analysis. Heatmaps were created using the R package “pheatmap” with Euclidean distances and hierarchical clustering for data visualization.

### Gene set enrichment analysis (GSEA)

Gene set enrichment analysis (GSEA) is a computational approach used to assess whether predefined gene sets show significant concordant differences between biological states^23^. The R package “clusterProfiler” (version 4.2.2) was used to conduct GSEA; all genes were ranked by their log2Fold Change values, and 1,000 gene set permutations were executed. In this study, the c2.cp.kegg.v7.5.1.symbols gene set, obtained from the Molecular Signatures Database (MSigDB), was used as a reference^23–25^. Gene sets with *P* < 0.05 were considered significantly enriched. The subsequent pathway analysis data were obtained from the KEGG database developed by Kanehisa Laboratories^26^, and this study has been authorized for use.

### Weighted gene coexpression network analysis (WGCNA) and identification of significant modules

The WGCNA algorithm was implemented using the R package “WGCNA” (version 1.70-3) to construct coexpression networks^27^. Pearson correlation coefficients were used to evaluate similarities in gene expression profiles, and correlation weights were transformed using a power function to establish a scale-free network topology. Using the “pickSoftThreshold” function, the coexpression similarity was increased to a power value (β = 2) to generate weighted adjacency matrices. Gene modules were defined as densely interconnected gene clusters in the coexpression network. Dynamic tree cutting in hierarchical clustering was used to identify distinct modules by converting adjacency matrices into topological overlap matrices for cluster analysis. Pearson correlation analysis was used to associate module eigengenes (MEs), which represent the first principal component and overall expression pattern of each module, with autophagy-lipid metabolism traits to identify significant module-trait relationships. Coexpression module structures were visualized through topological overlap heatmaps. A hierarchical clustering dendrogram of eigengenes combined with eigengene relationship heatmaps summarized the inter-module correlations. Differentially expressed genes (DEGs) related to autophagy and lipid metabolism were identified by intersecting DEGs with genes from the autophagy-lipid metabolism-associated module.

### GO term enrichment analysis

Gene Ontology (GO) analysis is a common approach for large-scale functional enrichment studies. GO annotation analysis of DEGs related to autophagy‒lipid metabolism (*P* < 0.05) was conducted using the R package “clusterProfiler” (version 4.2.2)^28^. Enrichment analysis was performed using overrepresentation analysis (ORA) based on the hypergeometric test to assess the significance of functional or pathway enrichment. The false discovery rate (FDR) was controlled by applying the Benjamini–Hochberg (BH) method for multiple testing corrections. The background gene list, defined as all detectable genes that passed expression filtering and were included in the differential analysis, served as the statistical background (universe) in the enrichment analysis.

### GeneMANIA

The GeneMANIA web platform (http://genemania.org) facilitates the prediction of functional associations among genes, including hub genes, through various interactions, such as protein‒protein interactions, protein‒DNA interactions, pathways, physiological/biochemical reactions, coexpression, and colocalization^29^. The GeneMANIA interface was employed to construct a PPI network for the primary candidate genes. In this study, genes from *Homo sapiens were analysed*. The screened list of key genes was submitted to the GeneMANIA online platform, and all analyses were performed using its default parameters and data settings. The default configuration automatically selects the most informative association networks to efficiently construct interaction networks and calculate the strengths of the associations among genes.

### Development and verification of diagnostic nomograms

In this study, a diagnostic diabetes nomogram model was developed using the R package “rms”. Risk scores were calculated on the basis of the expression levels of key genes, with total risk scores defined as the summations of individual gene risk scores. Calibration and receiver operating characteristic (ROC) curves were used to assess the diagnostic effectiveness of the diabetes nomogram.

### Receiver operating characteristic curve (ROC)

The receiver operating characteristic (ROC) curve is a valuable tool for assessing diagnostic test performance. ROC curves graphically illustrate the trade-off between sensitivity and specificity, serving as a comprehensive indicator of their continuous relationship. The area under the curve (AUC), which is based on cumulative sensitivity–specificity characteristics, is the predominant evaluation metric. In this study, the R package “pROC” was used to construct ROC curves, AUC values were calculated for feature gene screening, and the diagnostic accuracy was evaluated^30^. The area under the curve (AUC) values ranged from 0.5 to 1, with values near 1 indicating excellent diagnostic performance.

### Enzyme-linked immunosorbent assay (ELISA)

Serum samples were collected from 22 nondiabetic controls and 42 diabetic patients between February 25 and March 3, 2025, at the First Affiliated Hospital of the University of Science and Technology of China. On the basis of the kit specifications, standard curve preparation, and number of replicates needed, 20 samples were randomly selected from each of the control and diabetic groups for analysis using human EGR2 and CCR1 colorimetric ELISA kits (Novus Biologicals, USA; Cat# NBP3-08108/NBP2-75112). After collection, whole blood samples were allowed to clot at room temperature for 2 h or overnight at 2–8 °C, followed by centrifugation at 1000 × g for 15 min at 2–8 °C. Following centrifugation and aliquoting, the serum samples were immediately stored at -80 °C, and detections were performed within 15 days in strict accordance with the manufacturer’s instructions. This study was conducted in accordance with the Helsinki Declaration, and all participants provided informed consent forms. The Medical Research Ethics Committee of the First Affiliated Hospital of the University of Science and Technology of China approved this study, with ethics approval number 2025KY-075.

### Evaluation of diabetic mice models

Ten male mice, which consisted of five BKS-db and five age-matched BKS wild-type (6–8 weeks old) mice, were obtained from GemPharmatech Co., Ltd. (China). Following a 3-week acclimation period, endpoint random blood glucose levels were measured, followed by fasting blood glucose evaluations after 5–6 h of fasting. All the mice were humanely euthanized via the cervical dislocation method for terminal blood collection. Serum samples were separated and aliquoted (≥ 45 µl) for lipid profiling, encompassing HDL-C, LDL-C, TC, and TG measurements. Liver tissues were harvested, with portions either fixed for histology or cryopreserved at -80 °C. In addition, CRISPR/Cas9 technology was used to modify the *CCR1* gene (the second exon region) to construct *CCR1*-/- mice. The following is the gRNA sequence: 5S1: GCGCAAGACCTTAGAAGCGA and 3S1: ATAGAGGCACCAGGCATACC. The study was performed in accordance with the ARRIVE (Animal Research: Reporting of In Vivo Experiments) guidelines. This study was approved by the Animal Welfare and Ethics Review Committee of Wannan Medical College (approval number: WNMC-AWE-2024434), and all the experimental methods followed the relevant guidelines and regulations.

### Oil red O staining

Five liver tissue samples from BKS-db and BKS wild-type mice were analysed separately. Frozen Sects.  (4–5 μm thick) were prepared and processed as follows: they were rinsed with PBS, stained with Oil Red O solution (Sigma, USA; Cat# 00625) for 2–5 min, washed for 1–3 min, counterstained with haematoxylin (Nanjing Jiancheng Bioengineering Institute, China; Cat# D005) for 1–3 min, rinsed again for 1–3 min, and mounted using glycerol gelatine (Sinopharm Chemical Reagent Co., Ltd., China; Cat# 10004160). Reagents: xylene (Sinopharm Chemical Reagent Co., Ltd., China; Cat# 1330-20-7); methanol (Sinopharm Chemical Reagent Co., Ltd., China; Cat# 67-56-1); ethanol (Sinopharm Chemical Reagent Co., Ltd., China; Cat# 64-17-5); immunohistochemistry pen (Nanjing KGI Biotechnology, China; Cat# KGSP10); PBS buffer (Nanjing KGI Biotechnology, China; Cat# KGB5001), and a high-temperature resistant slide rack (Maxim Biotech, China; Model# RAK-2001).

### Western blot

Liver tissues from mice were lysed in RIPA lysis buffer supplemented with PMSF (RIPA: PMSF = 100:1, v/v). The lysates were collected and centrifuged at 12,500 × g for 10 min. Protein concentrations were measured with a NanoDrop 2000 microplate spectrophotometer (Thermo Fisher Scientific, USA). The proteins were separated via SDS‒PAGE (12% gel) and transferred onto PVDF membranes. The membranes were blocked using 5% skim milk at room temperature for 2 h and then incubated overnight with primary antibodies at 4 °C. The membranes were then incubated with the appropriate secondary antibodies at room temperature for 2 h. Protein signals were detected using ECL reagent and an imaging system. Protein expressions were semiquantitatively analysed using ImageJ software (version 1.52). The antibodies used were against β-actin (ABclonal Technology, China; Cat# AC038); CCR1 (Thermo Fisher Scientific, USA; Cat# PA1-41062); EGR2 (ABclonal Technology, China; Cat# A3219); P62 (ABclonal Technology, China; Cat# A19700); and LC3B (Abcam Technology; Cat# ab192890)^31^.

### Total RNA preparation and quantitative real-time PCR

Following the manufacturer’s guidelines, total RNA was isolated from mouse liver tissue using TRIzol reagent (Invitrogen, USA). RNA concentrations and purities were assessed with a NanoDrop 2000 spectrophotometer (Thermo Scientific, USA). Using the RevertAid First Strand cDNA Synthesis Kit (Thermo Scientific, USA), 1 µg of total RNA was reverse transcribed into cDNA according to the manufacturer’s instructions. SYBR Green Master Mix (11184ES03; Yeasen, China) was used for quantitative real-time PCR on a QuantStudio 5 Real-Time PCR System (Applied Biosystems, USA). The thermal cycling protocol included initial denaturation at 95°C for 30 seconds, followed by 40 cycles of 5 seconds of denaturation at 95°C and 30 seconds of annealing/extension at 60°C. Each sample was analysed in triplicate. Gene expression levels were determined using the 2^−ΔΔCt method, with β-actin used as the reference gene. The following primer sequences were used: ACTIN-F: 5’-GGACTCCTATGTGGGTGACG-3’; ACTIN-R: 5’-CTTCTCCATGTCGTCCCAGT-3’; P62-F: 5’-GCACAGGCACAGAAGACAAG-3’; and P62-R: 5’-CCACCGACTCCAAGGCTAT-3’^31^.

### Statistical analysis

Statistical analyses were conducted using R software (version 4.1.2). Spearman’s correlation test was employed to assess associations among variables. The Wilcoxon test was used to analyse the differences between two groups, and the Kruskal‒Wallis test was employed for comparisons among three or more groups. Statistical significance was defined as a two-tailed P value less than 0.05.

## Results

### Diabetes-related DEGs

**Fig. 1:**
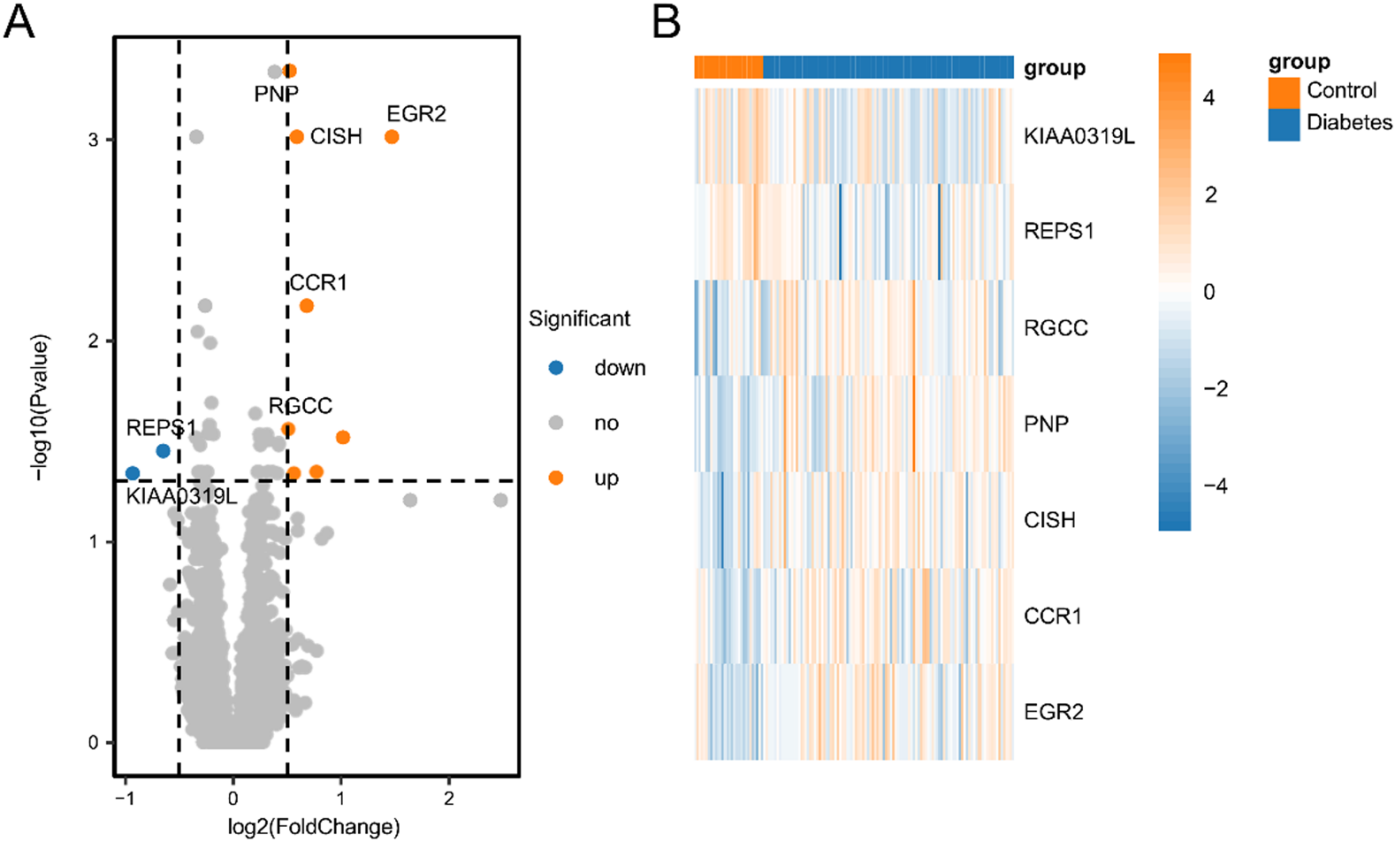
Analysis of diabetes-related DEGs. (**A**) Volcano plot illustrating the distributions of differentially expressed genes (DEGs) between the diabetes and control groups. The grey dots represent genes whose expressions did not significantly differ; and (**B**) Heatmap showing the top-ranked DEGs.

A comparative analysis between the diabetic and control samples revealed 10 DEGs whose expressions significantly differed (*P* < 0.05, |log2-fold change| > 0.5). In the diabetic samples, 8 genes were upregulated, and 2 were downregulated (Table S3). All DEGs were visualized in a volcano plot (Fig. 1A), while the expression patterns of the top-ranked genes (e.g., *RGCC*,* PNP*,* CISH*,* CCR1*,* EGR2*,* KIAA0319L*,* and REPS1*) across samples are illustrated in a heatmap (Fig. 1B).

### Construction of weighted gene coexpression networks and identification of modules

**Fig. 2:**
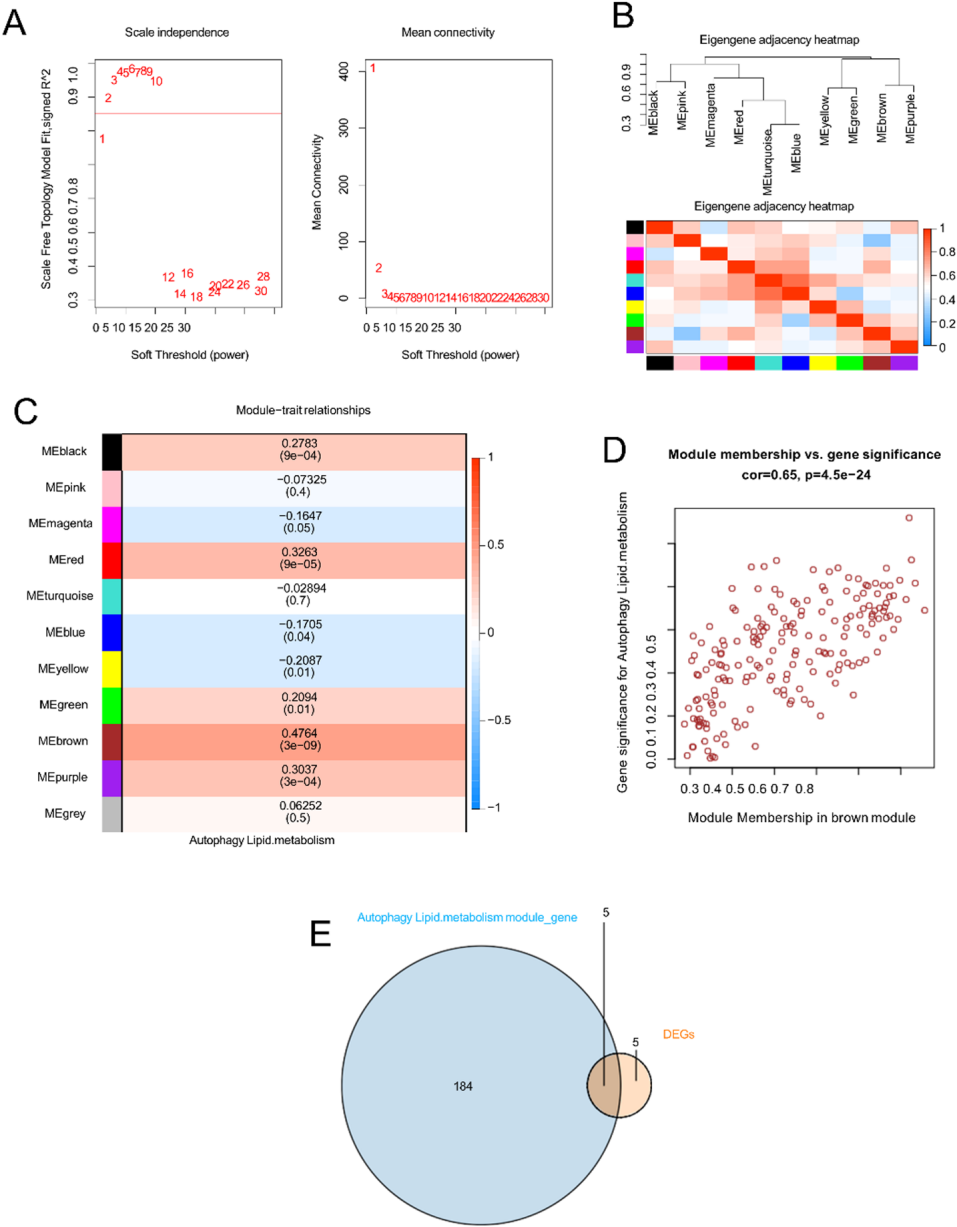
Construction of the WGCNA coexpression network. (**A**) Soft threshold power (β = 2) and scale-free topology fit index (R²); (**B**) module associations. Correlation heatmaps of eigengene networks. Each row and column in the heatmap represent colour-coded module eigengenes. Red signifies high adjacency, whereas blue represents low adjacency. Red squares on the diagonal indicate meta-modules; (**C**) Consensus module eigengene-trait correlations with autophagy and lipid metabolism. Each consensus module is represented by a row, while each trait is represented by a column. The correlation coefficients between module eigengenes and traits are presented, with corresponding P values indicated in brackets. Correlations are visually represented using colour codes as per the legend; (**D**) The correlation between module membership (MM) and gene significance (GS) for autophagy and lipid metabolism within the brown module is displayed. Cor represents the absolute correlation coefficient between GS and MM; and (**E**) Venn diagram illustrates the overlap of autophagy- and lipid metabolism-related module genes with DEGs.

WGCNA was applied to study gene sets associated with autophagy-lipid metabolism. Analyses of scale independence and mean connectivity indicated that a soft threshold of β = 2 resulted in near-zero mean connectivity and scale independence > 0.85 (Fig. 2A). Module eigengenes (Mes) were correlated to investigate intermodule relationships, with eigengene networks visualized via a heatmap (Fig. 2B). On the basis of the module-trait correlation heatmap (Fig. 2C), genes that clustered in the brown module (*n* = 189; Table S4) exhibited the strongest positive correlations with autophagy-lipid metabolism (*r* = 0.4764; *P* < 0.05). Consequently, the brown module was prioritized for subsequent analyses because of its potential to accurately reflect autophagy-lipid metabolism. A scatterplot illustrating the relationship between gene significance (GS) and module membership (MM) for autophagy-lipid metabolism within the brown module is shown in Fig. 2D. A significant positive correlation (*cor* = 0.65, *P* < 0.05) between MM and GS suggests a strong association of the central hub genes in this module with autophagy-lipid metabolism. Intersection analysis revealed five autophagy-lipid metabolism-related DEGs that were shared between the DEGs and module genes (Table S5, Fig. 2E).

### Machine learning algorithms for screening key hub genes

**Fig. 3:**
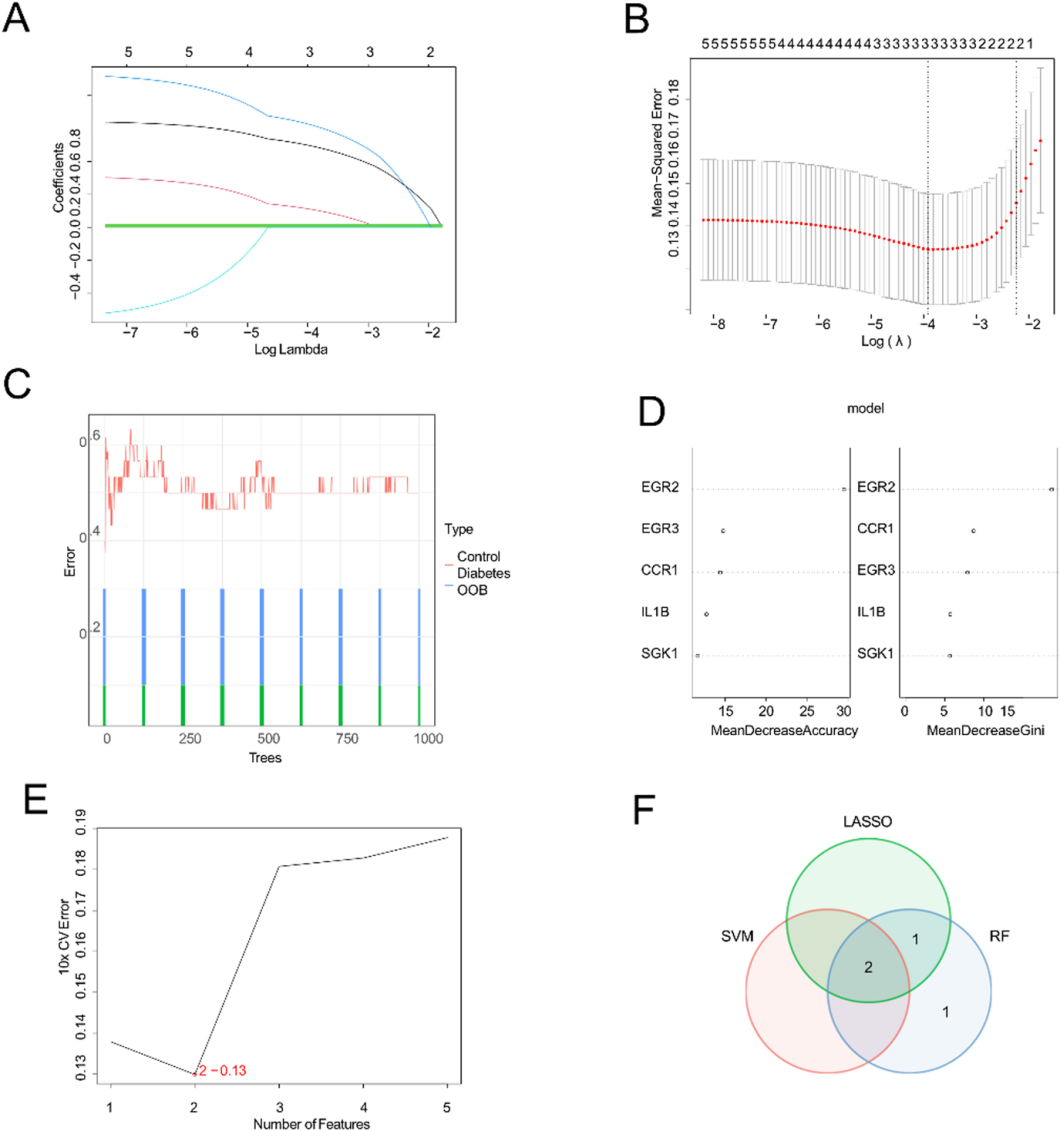
Identification of potential diagnostic biomarkers for diabetes through machine learning methods. (**A**) LASSO regression trajectory of variable coefficients; (**B**) confidence intervals for each lambda in LASSO regression; (**C**) random forest error rates compared to the numbers of classification trees; (**D**) top 4 autophagy + lipid metabolism-related DEGs ranked by two importance metrics in the random forest algorithm; (**E**) identification of optimal feature genes using SVM-RFE; and (**F**) Venn diagram showing the intersection of three machine learning algorithms.

This study further employed LASSO regression and random forest algorithms to identify key hub genes. LASSO regression analysis revealed three key differentially expressed genes (DEGs) related to autophagy and lipid metabolism: *EGR2*, *IL1B*, and *CCR1* (Fig. 3A, B). The random forest algorithm identified the top four genes as key differentially expressed genes (DEGs) related to autophagy and lipid metabolism using feature importance metrics, such as the mean decrease in accuracy (MDA) and mean decrease in Gini (MDG) (Fig. 3C, D). Support vector machine (SVM) analysis revealed two autophagy- and lipid metabolism-related DEGs (e.g., *EGR2* and *CCR1*) (Fig. 3E). Intersection of the DEGs that were identified by all three methods revealed two core hub genes (e.g., *EGR2* and *CCR1*) for subsequent analyses (Fig. 3F).

### The expressions of the hub genes and single-gene GSEA enrichment

**Fig. 4:**
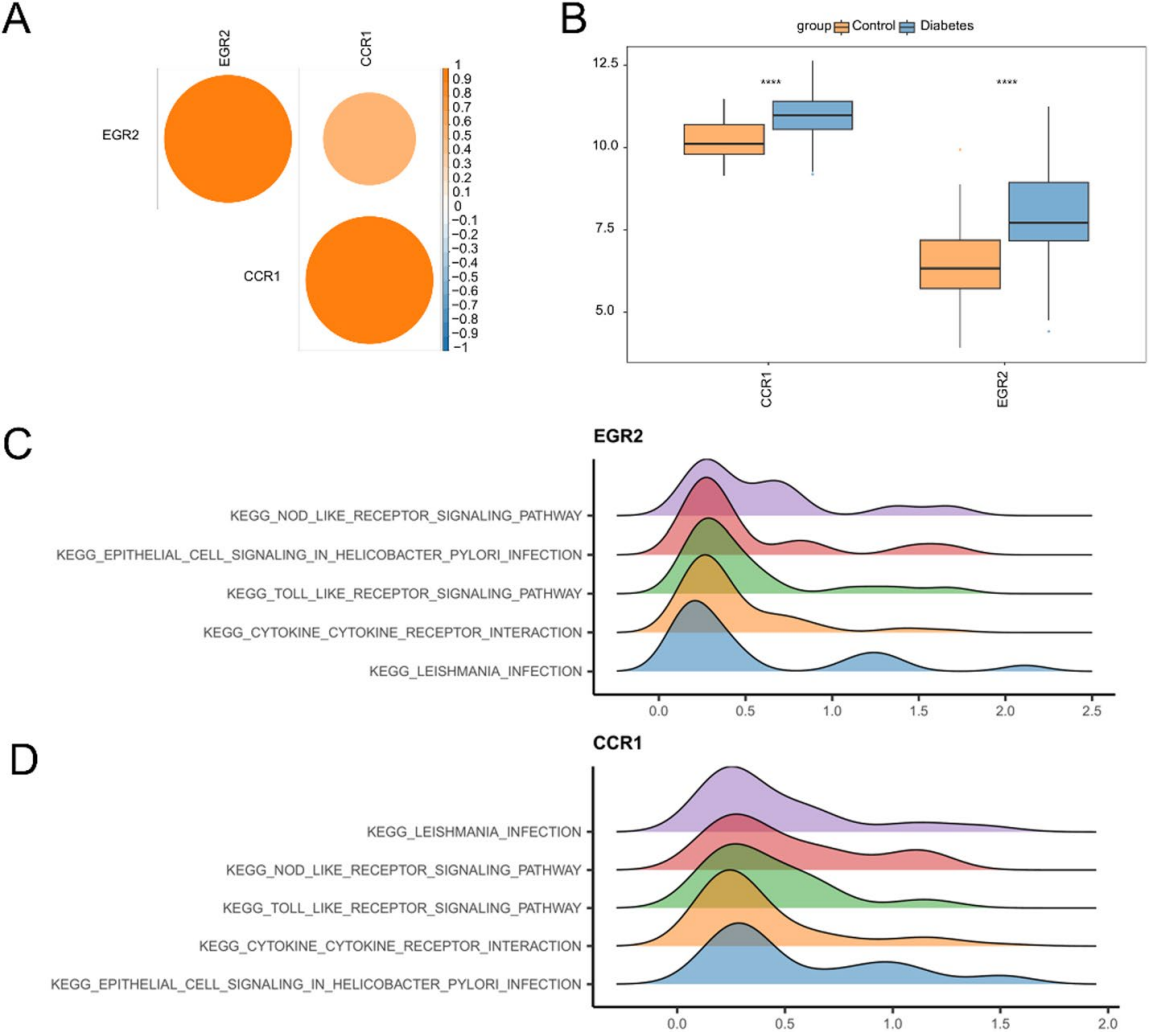
Expressions of the hub genes and single-gene GSEA enrichment. (**A**) Heatmap illustrating hub gene correlations; (**B**) Box plots depicting hub gene expression levels; (**C**) GSEA for EGR2; and (**D**) GSEA for CCR1.

In this study, the expression patterns of the hub genes in the diabetic and control groups were examined via correlation heatmaps and box plots. A strong positive correlation was noted between the two hub genes (Fig. 4A). The results of the rank-sum test revealed significantly higher expression levels of *EGR2* and *CCR1* in the diabetic group than in the control group (Fig. 4B). The authors conducted a single gene GSEA to investigate the mechanisms through which hub genes impact diabetes (Fig. 4C, D). Genes exhibiting expression patterns akin to those of *EGR2* were enriched primarily in pathways, such as *KEGG_LEISHMANIA_INFECTION*, *KEGG_CYTOKINE_CYTOKINE_RECEPTOR_INTERACTION*, *KEGG_TOLL_LIKE_RECEPTOR_SIGNALING_PATHWAY*, *KEGG_EPITHELIAL_CELL_SIGNALING_IN_HELICOBACTER_PYLORI_INFECTION*, and *KEGG_NOD_LIKE_RECEPTOR_SIGNALING_PATHWAY*. Genes linked to CCR1 expression were enriched in *KEGG_EPITHELIAL_CELL_SIGNALING_IN_HELICOBACTER_PYLORI_INFECTION*, *KEGG_CYTOKINE_CYTOKINE_RECEPTOR_INTERACTION*, *KEGG_TOLL_LIKE_RECEPTOR_SIGNALING_PATHWAY*, *KEGG_NOD_LIKE_RECEPTOR_SIGNALING_PATHWAY*, and *KEGG_LEISHMANIA_INFECTION*.

### Hub gene interaction analysis

**Fig. 5:**
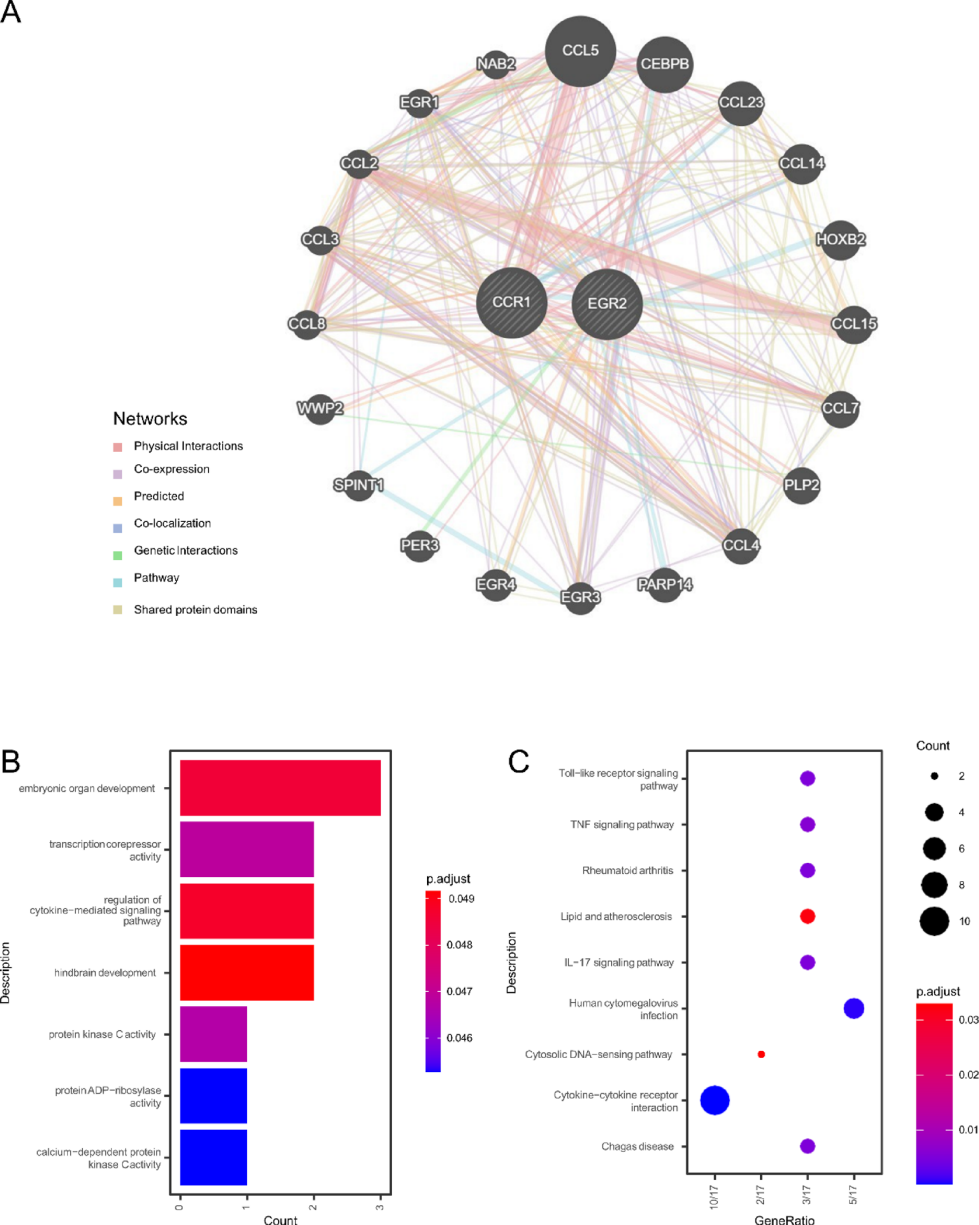
Interaction analysis of the hub genes. (**A**) Hub gene coexpression network; (**B**) GO analysis of coexpressed genes; and (**C**) KEGG analysis of coexpressed genes.

Utilizing the GeneMANIA database, a protein‒protein interaction (PPI) network was constructed in this study to elucidate the interactions between two hub genes (Fig. 5A). GO and KEGG analyses were conducted on 22 genes, which consisted of 2 hub genes and 20 interacting genes, to explore their functional roles. GO enrichment analysis revealed significant enrichment in biological processes (BP), such as embryonic organ development (GO:0048568), regulation of cytokine-mediated signalling (GO:0001959), and hindbrain development (GO:0030902). Additionally, molecular functions (MF) terms included calcium-dependent protein kinase C activity (GO:0004698), protein ADP-ribosylase activity (GO:1990404), protein kinase C activity (GO:0004697), and transcription corepressor activity (GO:0003714) (Fig. 5B, Table S6). KEGG pathway analysis revealed predominant enrichment of viral protein interaction with cytokine and cytokine receptor (has04061), chemokine signaling pathway (has04062), cytokine-cytokine receptor interaction (has04060), human cytomegalovirus infection (has05163), rheumatoid arthritis (has05323), IL-17 signaling pathway (has04657), Chagas disease (has05142), Toll-like receptor signaling pathway (has04620), and TNF signaling pathway (has04668) (Fig. 5C, Table S7).

### Diagnostic value of the hub genes

**Fig. 6:**
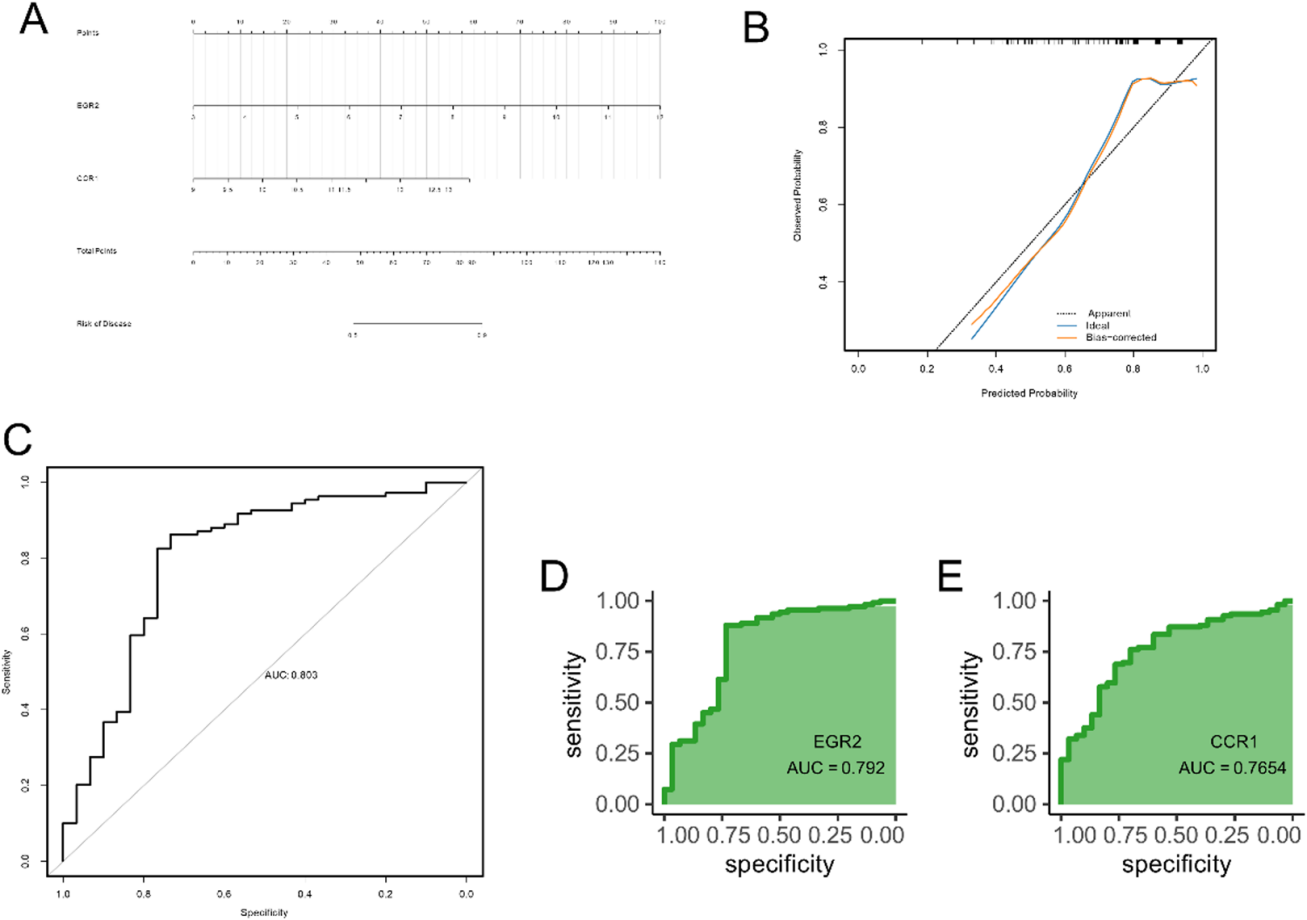
Construction and validation of the diagnostic diabetes nomogram model and ROC curves of the hub genes. (**A**) Nomogram for diabetes prediction; (**B**) calibration curve for evaluating the predictive performance of the nomogram; (**C**) ROC curve for assessing the clinical utility of the nomogram; (**D**) ROC curve for EGR2; and (**E**) ROC curve for CCR1.

A diagnostic diabetes nomogram model was developed utilizing the hub genes *EGR2* and *CCR1* (Fig. 6A), and its predictive performance was evaluated with a calibration curve. The calibration curve demonstrated minimal discrepancies between the actual and predicted diabetes risks, indicating high model accuracy (Fig. 6B). ROC curve analysis further confirmed the validity of the model (Fig. 6C). ROC analysis demonstrated the diagnostic potential of the hub genes, with *EGR2* and *CCR1* achieving AUC values of 0.792 and 0.765, respectively, both surpassing 0.6 (Fig. 6D, E), suggesting their discriminative capacity as potential biomarkers for diabetes.

### The expression levels of CCR1 and EGR2 in the serum of diabetic patients and the liver of db mice significantly increased

**Fig. 7:**
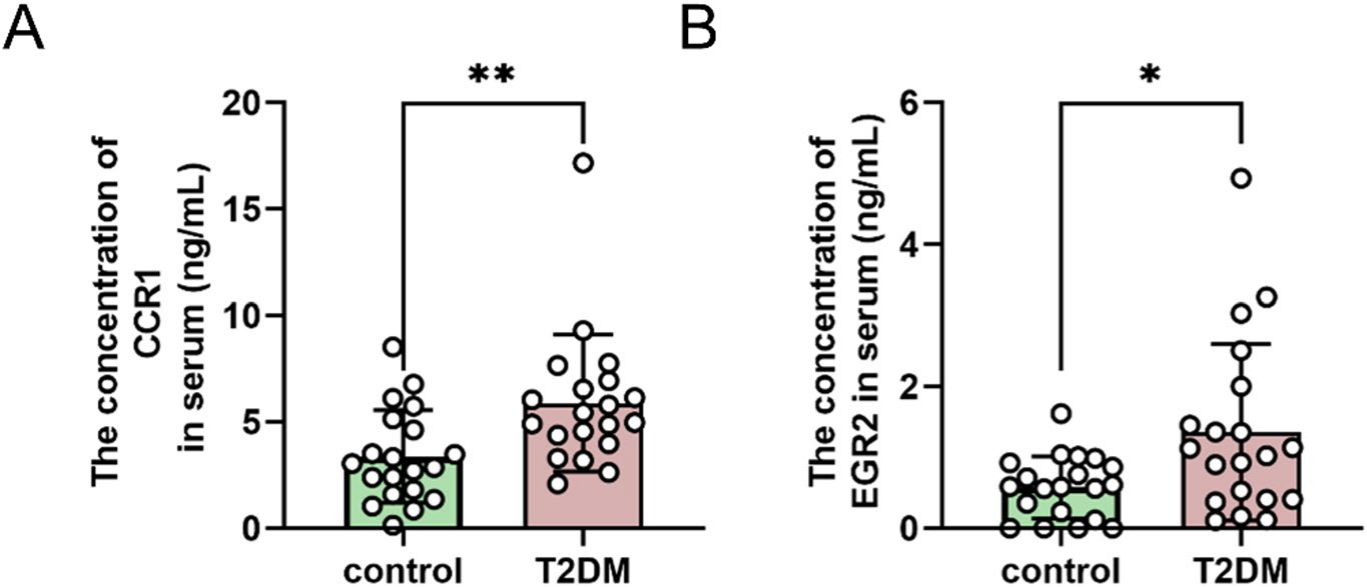
The expressions of CCR1 and EGR2 in human serum samples. (**A**) ELISA was used to detect the expression of CCR1 in human serum; and (**B**) ELISA was used to detect the expression of EGR2 in human serum.

**Fig. 8:**
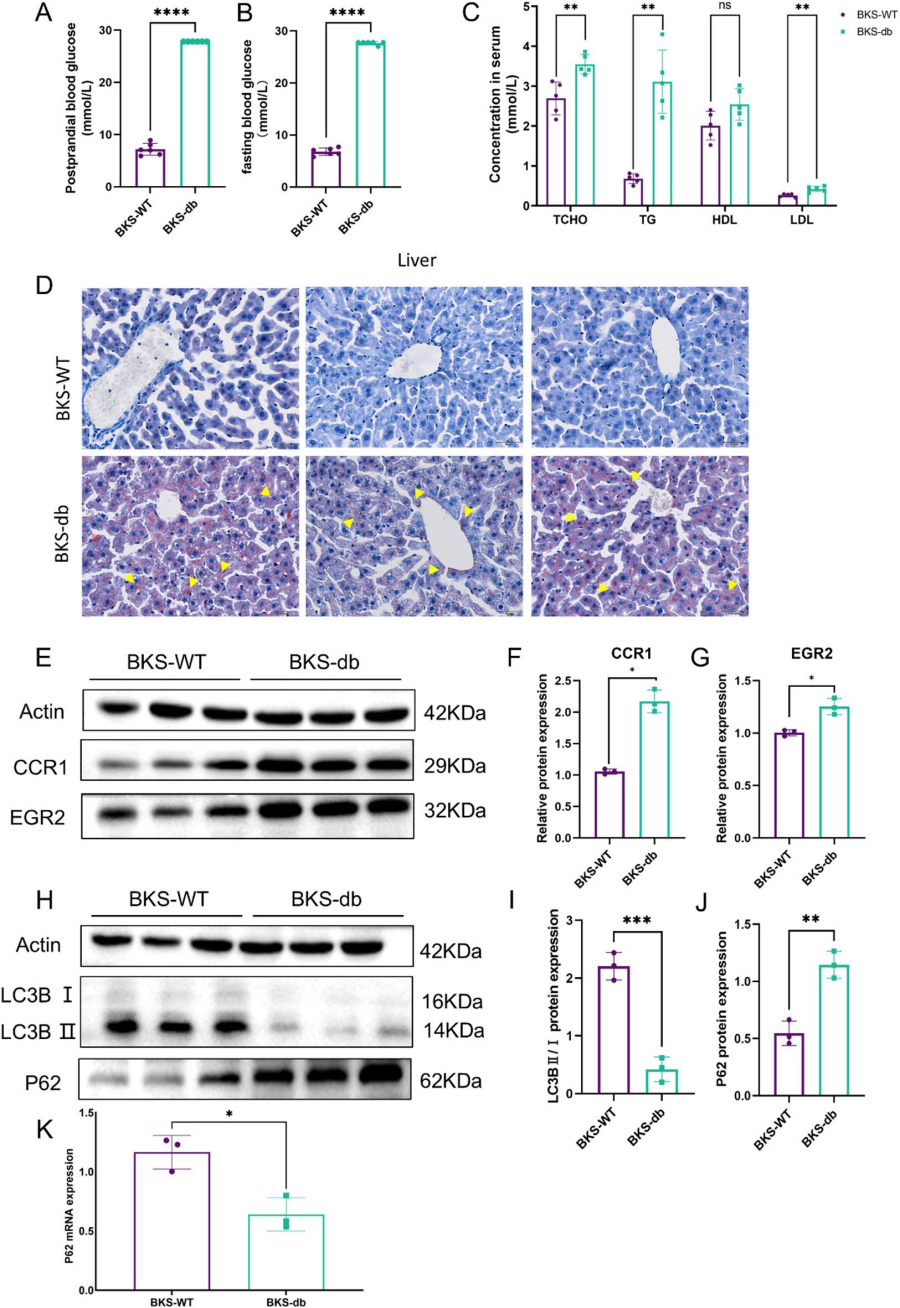
The expressions of CCR1 and EGR2 in the livers of db mice were significantly increased, and autophagic flow was blocked. (**A**) Postprandial blood glucose levels in mice; (**B**) fasting blood glucose levels in mice; (**C**) serum levels of TCHO, TG, HDL, and LDL in mice; (**D**) Oil red O staining of liver tissues in mice; (**E**–**G**) Western blot analysis of CCR1 and EGR2 expressions in liver tissues with statistical results; (**H**–**J**) Western blot analysis of LC3B and P62 expressions in liver tissues with statistical results; and (**K**) the mRNA level of P62.

To further validate the differential expressions of CCR1 and EGR2 in diabetes, human serum samples (the baseline characteristics are shown in Table 1) were collected and analysed using ELISA kits. The results revealed significantly elevated expression levels of CCR1 and EGR2 in the diabetic group compared with those in the control group (Fig. 7A, B; *P < 0.05, **P < 0.01). Serum biochemical parameters were measured in 6–8-week-old male BKS wild-type and BKS-db mice. Compared with wild-type mice, BKS-db mice exhibited significantly higher postprandial random blood glucose, fasting blood glucose, LDL, TCHO, and TG levels, with no significant difference in HDL levels (Fig. 8A–C; ns *P* > 0.05, *P < 0.05, ***P < 0.0001). Liver tissues from mice were subjected to Oil Red O staining and Western blot analysis. BKS-db mice showed marked lipid accumulations in liver tissues (Fig. 8D). Compared with those in wild-type mice, the CCR1 and EGR2 expression levels in BKS-db mice were significantly upregulated in both tissues (Fig. 8E–G, *P* < 0.05), which is consistent with the results of the bioinformatics analysis. In addition, in the livers of db mice, there was a significant decrease in the transformation of LC3B, a decline in the mRNA level of P62, and a significant increase in the protein level (Fig. 8H–K), suggesting that autophagic degradation was impaired and that autophagic flow was blocked in their livers.

**Table 1:** Basic information for the two groups.

Variables	Total (*n* = 64)	normal (*n* = 22)	Diabetes (*n* = 42)	Statistic	*P*
Age, Mean ± SD	54.83 ± 12.93	47.45 ± 10.44	58.69 ± 12.52	t=-3.60	**< 0.001**
GHB (total glycated haemoglobin%), M (Q₁, Q₃)	7.25 (6.39, 8.55)	6.25 (6.12, 6.48)	8.23 (7.56, 9.16)	Z=-5.95	**< 0.001**
HbA1c, M (Q₁, Q₃)	6.75 (5.73, 8.15)	5.70 (5.45, 5.80)	7.70 (6.80, 8.62)	Z=-5.60	**< 0.001**
Fasting blood glucose, M (Q₁, Q₃)	6.18 (5.15, 7.86)	5.14 (4.75, 5.25)	7.26 (6.16, 8.93)	Z=-5.12	**< 0.001**
TG, M (Q₁, Q₃)	1.25 (0.90, 2.07)	1.11 (0.89, 1.69)	1.44 (0.98, 2.28)	Z=-1.21	0.225
VLDL-C, M (Q₁, Q₃)	0.68 (0.57, 0.95)	0.66 (0.56, 0.85)	0.70 (0.57, 0.95)	Z=-0.77	0.441
Creatinine µmol/l, M (Q₁, Q₃)	57.00 (48.50, 72.50)	59.00 (49.25, 72.50)	57.00 (48.00, 72.00)	Z=-0.49	0.621
Gender (1 = male, 2 = female), n (%)				χ²=1.22	0.269
male	38 (59.38)	11 (50.00)	27 (64.29)		
female	26 (40.62)	11 (50.00)	15 (35.71)		

### The increase in blood glucose levels under high-fat conditions was effectively inhibited *in CCR1-/-* mice

**Fig. 9:**
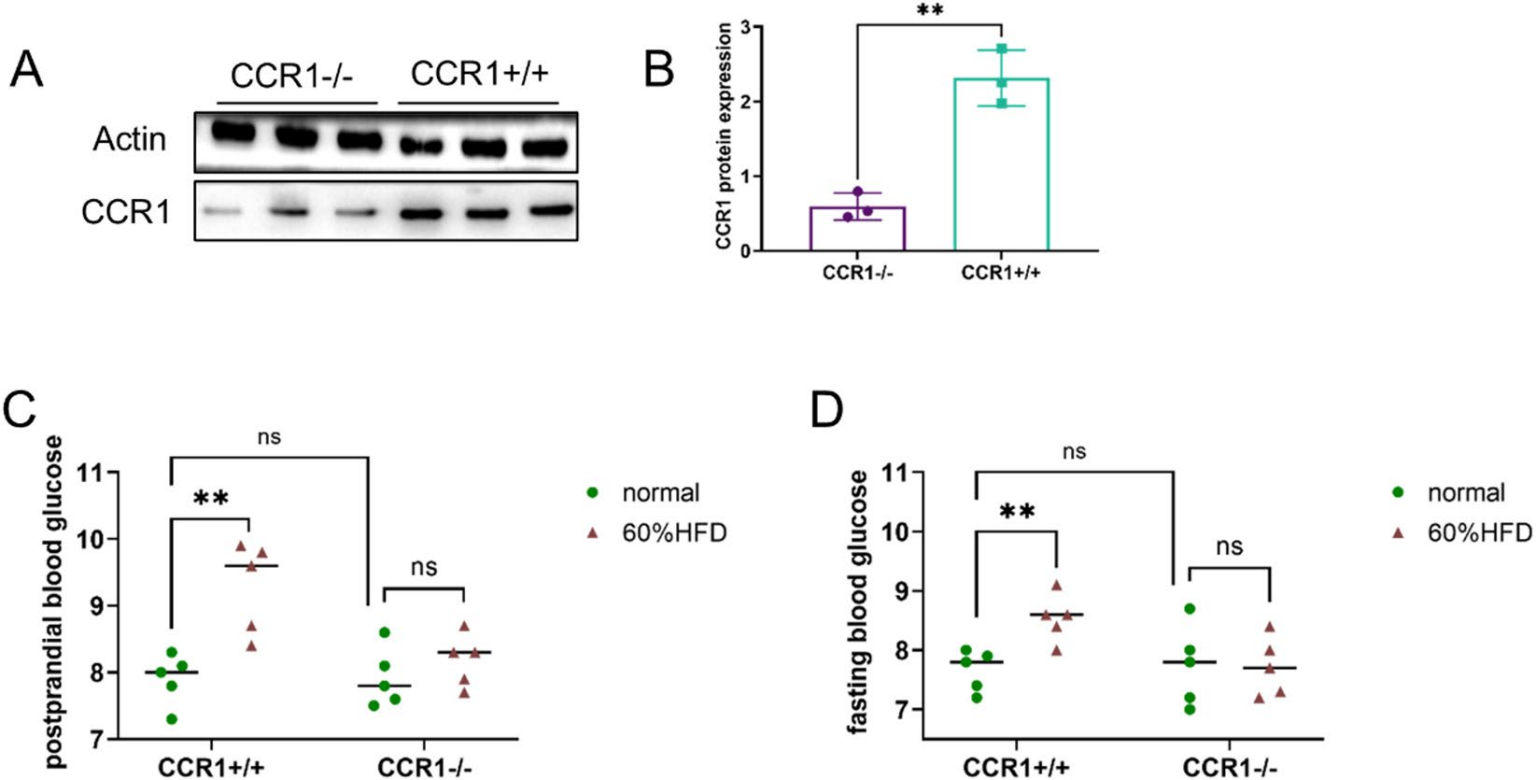
Verification of CCR1-/- mice and observation of blood glucose levels under high-fat conditions. (**A**,**B**) Western blot analysis and statistical evaluation of CCR1 expression in liver tissues; (**C**) postprandial blood glucose levels in mice; and (**D**) fasting blood glucose levels in mice.

These findings suggest that CCR1 and EGR2 could serve as potential biomarkers for diabetes. To further explore the correlation between them, the authors obtained *CCR1* gene knockout mice and verified them (Fig. 9A, B, **P < 0.01) and then observed the changes in blood glucose levels through high-fat feeding (for a period of 3 weeks, consistent with the time period of the mice mentioned earlier). The grouping was as follows: *CCR1 +/+*, 60% HFD *CCR1 +/+*, *CCR1 -/-*, and 60% HFD - *CCR1 -/-*. Compared with the CCR1+/+ group, the 60%HFD-CCR1+/+ group presented significantly higher postprandial and fasting blood glucose levels. Under standard feeding conditions, postprandial and fasting blood glucose levels were similar between the *CCR1*+/+ and *CCR1*-/- groups. Notably, compared with those in the normal feeding group, the blood glucose levels in the 60%HFD-*CCR1-/-* group did not significantly differ, indicating effective blood glucose control (Fig. 9C, D; ns *P* > 0.05, **P < 0.01).

## Discussion

Lipophagy is a selective autophagic process that involves the degradation of lipid droplets (LDs). Upon the activation of lipophagy, autophagosomes encapsulate LDs and fuse with lysosomes, enabling lipid degradation and recycling^32^. Dysregulation of this mechanism leads to pathological lipid accumulation. In diabetes and its complications, disruption of lipid metabolic homeostasis is recognized as a critical driving force for these conditions^33,34^, whereas stable lipid metabolism effectively ameliorates insulin resistance^35,36^. However, more extensive research on diabetes has focused only on general autophagy activity or the autophagic flux phenotype, such as related research on the AMPK signalling pathway^37–39^. This limited scope hinders the comprehensive exploration of diabetic pathogenesis and the development of targeted therapies. Furthermore, existing research has yet to clarify the causal relationship between lipid metabolism dysregulation and diabetes, often attributing their interaction to bidirectional causality. Thus, the identification of more precise biomarkers may unlock novel research avenues to elucidate these mechanisms.

In this study, various bioinformatics methods, such as differentially expressed gene (DEG) analysis and weighted gene coexpression network analysis (WGCNA), were used to identify genes linked to diabetes. The results revealed associations between lipophagy and diabetic mechanisms, particularly between *EGR2* and *CCR1*, as determined by machine learning algorithms (Fig. 3). These predictions were verified by the ELISA results, which revealed that CCR1 and EGR2 were significantly upregulated in the serum samples of diabetic patients (Fig. 7). In addition, compared with those in wild-type mice, the expression levels of CCR1 and EGR2 in the livers of BKS-db mice were significantly greater (Fig. 8). This upregulation may reflect the adaptive mechanism of cells to metabolic imbalance in the diabetic state, although the dysregulation of these genes may also actively promote pathological progression. *CCR1* and *EGR2* clearly have great potential as biomarkers for diabetes diagnoses. By constructing precise diagnostic models and conducting receiver operating characteristic (ROC) curve analyses, the areas under the curve (AUC) for both models were greater than 0.6 (Fig. 6). To further support the relationship between CCR1 and diabetes (or abnormal glycolipid metabolism), a *CCR1-/-* mouse model was constructed in this study. These findings indicate that under standard feeding conditions, *CCR1*-/- mice and normal mice exhibit comparable blood glucose levels, suggesting that *CCR1* gene knockdown alone does not impact blood glucose levels. A high-fat diet significantly increased postprandial and fasting blood glucose levels in normal mice, but this phenomenon was absent in *CCR1*-/- mice. Their blood glucose concentrations seem to have been effectively controlled and were not different from those of normally raised *CCR1-/-* mice (Fig. 9). These experimental results indicate that CCR1 is associated with blood glucose regulation and participates in blood glucose regulation, at least in a high-fat environment.

CCR1 (C-C chemokine receptor type 1) is a key chemokine receptor that is extensively involved in immune cell migration and inflammatory responses^40,41^. While the specific association between CCR1 and diabetes remains unelucidated, chronic inflammation—a hallmark of diabetes—suggests a potential role for CCR1 in regulating immune cell trafficking and activation within this context^42,43^. Specifically, CCR1 expressions are correlated with diabetes-related pathological states, including insulin resistance, inflammatory responses, and microvascular complications^42,44,45^. Single-gene GSEA indicated that genes whose expression patterns were similar to those of *CCR1* were enriched primarily in the *TOLL_LIKE_RECEPTOR_SIGNALING_PATHWAY* (Fig. 4). Toll-like receptors (TLRs) play crucial roles in type 1 and type 2 diabetes, as well as diabetic complications^46–49^, primarily by influencing inflammation and immune dysregulation^50^, highlighting the long-term significance of investigating the role of TLR-CCR1 interplay in diabetes. Concurrently, lipid accumulation plays a pivotal role in diabetic pathogenesis, especially by driving inflammation. Excessive lipid deposition triggers the release of inflammatory factors (e.g., TNF-α and IL-6), exacerbating metabolic dysfunction and tissue damage. Adipose tissue expansion and dysfunction further amplify this cycle, promoting insulin resistance^7,51^. Chronic low-grade inflammation, tightly linked to lipid overload, is prevalent in individuals with type 2 diabetes and may be associated with its complications^52^. Autophagy, while protective in insulin-sensitive tissues and β-cells, is suppressed under lipid overload conditions, and worsening lipid accumulation and lipotoxicity from this imbalance significantly contribute to T2DM pathogenesis^53^, necessitating strategies to disrupt the vicious cycle of lipid autophagy. Lipophagy alleviates insulin resistance and β-cell damage by selectively degrading lipid droplets^54^, yet proinflammatory cytokines such as TNF-α and IL-6 suppress lipophagy, perpetuating lipid-driven inflammation^55^. In summary, *CCR1* is intricately linked to inflammation—a central diabetic feature—and intersects with lipid metabolism and autophagy. These findings position *CCR1* as a potential nexus mediating these interconnected processes. However, the relationship between CCR1 and lipid metabolism remains unclear, although indirect modulation via the gut‒liver axis or microbiota interactions is hypothesized^56,57^.

Another pivotal gene, *EGR2*, was shown in this study to be highly positively correlated with *CCR1* expression. Furthermore, GSEA enrichment and interaction analyses suggested a potential synergistic relationship between these two genes (Figs. 4 and 5). Previous research has shown that EGR2, like CCR1, is crucial for immune responses and the development of autoimmune diseases^58,59^. EGR2 has also been implicated in diabetes research, with one study showing that inhibiting EGR2 expression ameliorates high-fat diet-induced insulin resistance^60^. EGR2 significantly affects lipid metabolism, especially in metabolic disorders such as NAFLD and metabolic syndrome. Research has indicated that EGR2 regulates monocyte differentiation into liver lipid-associated macrophages and is critical in metabolic dysfunction-associated liver fibrosis^61^. Another study demonstrated that under insulin-resistant conditions, EGR2 induction promotes the upregulation of miR-455 expression to facilitate adaptive β-cell proliferation in mice^62^. These findings suggest that EGR2 may play dual roles in the initiation and progression of diabetes. Therefore, whether the above effects are mediated by a feedback mechanism and the specific interaction of EGR2/CCR1 in diabetes deserves further study.

Although the results of the present study preliminarily suggest the potential of EGR2 and CCR1 as candidate biomarkers related to diabetes, systematic future research is still needed to further establish clinical and mechanistic evidence. Specific research directions include the following: First, in multicentre clinical cohorts with expanded sample sizes, serum levels of EGR2/CCR1 and key indicators of lipophagy should be simultaneously measured, and their independent correlations with diabetes stage, metabolic parameters, and complications should be clarified through multivariate analyses. Second, utilizing diabetic animal models and cellular models, the specific regulatory effects of EGR2 and CCR1 on the lipophagy process via gene silencing/overexpression techniques should be elucidated. Third, spatial transcriptomics and proteomics technologies should be used to analyse the colocalization relationship between EGR2/CCR1 expressions and lipophagy activities in metabolic tissues such as the liver and adipose tissue, as well as their downstream signalling networks. These investigations will not only provide multilevel evidence supporting the use of EGR2 and CCR1 as diagnostic markers or therapeutic targets but also hold promise for identifying novel regulatory axes of lipophagy imbalance in the onset and progression of diabetes, offering new perspectives for the precise intervention of metabolic diseases.

## Supplementary Information

Below is the link to the electronic supplementary material.

Supplementary Material 1

Supplementary Material 2

Supplementary Material 3

Supplementary Material 4

Supplementary Material 5

Supplementary Material 6

Supplementary Material 7

Supplementary Material 8

Supplementary Material 9

## Data Availability

The datasets analysed during the current study are available in the Science Data Bank repository, https://doi.org/10.57760/sciencedb.28870.
