# Supplementary figures and images for: Identification and verification of the key genes, CCR1 and EGR2, in diabetes-associated lipophagy
Source: Sci Rep. 2026 Mar 20;16:14274. doi: 10.1038/s41598-026-43737-9 (PMC13139434; doi:10.1038/s41598-026-43737-9)

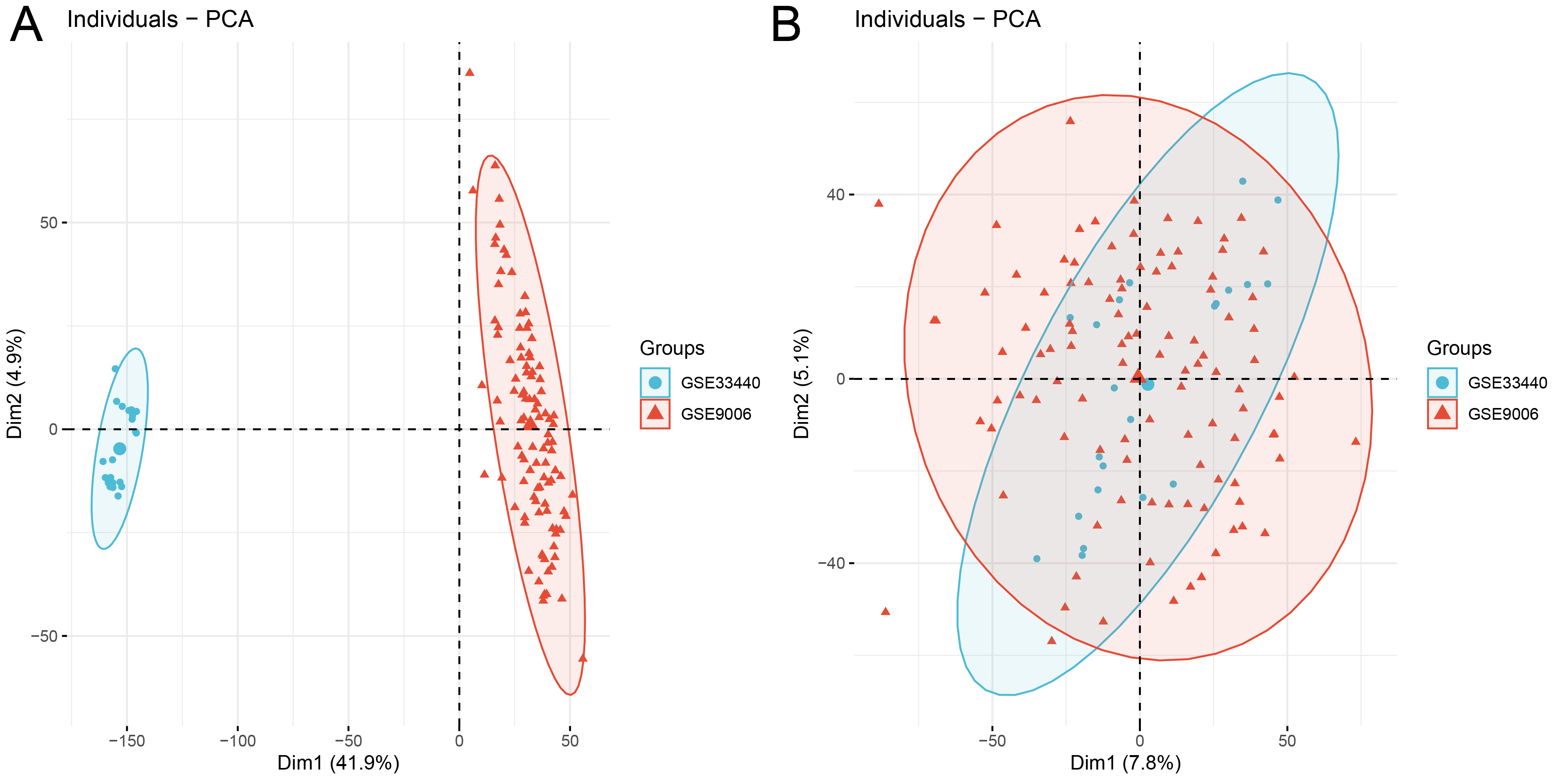

Supplement: Supplementary file 1 — Supplementary Material 1 [file 41598_2026_43737_MOESM1_ESM.tif]
