# Supplementary material for: Identification and verification of the key genes, CCR1 and EGR2, in diabetes-associated lipophagy
Source: Sci Rep. 2026 Mar 20;16:14274. doi: 10.1038/s41598-026-43737-9 (PMC13139434; doi:10.1038/s41598-026-43737-9)

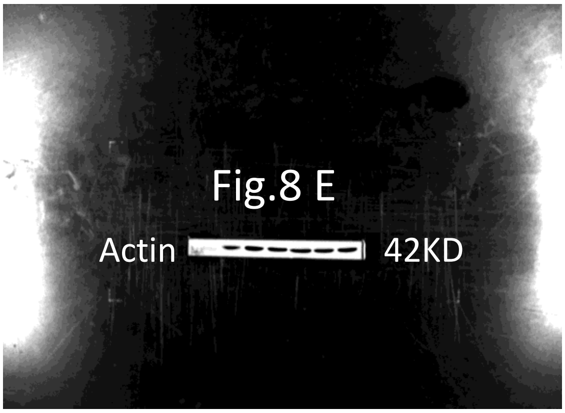
Fig.8 E
Actin
42KD

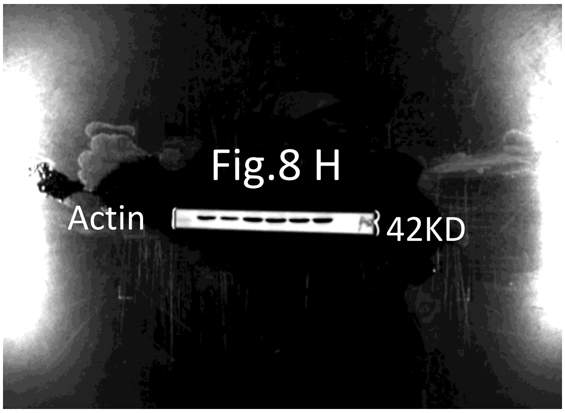
Fig.8 H
Actin
42KD

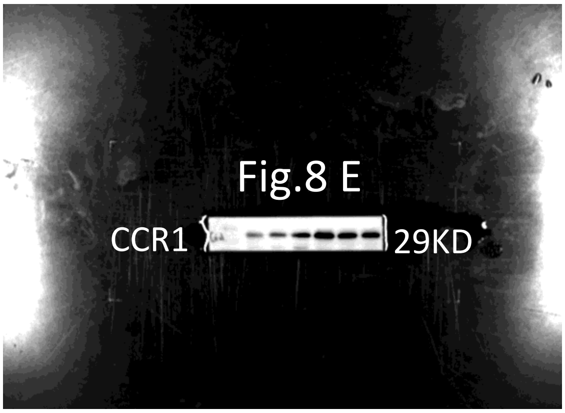
Fig.8 E
CCR1
29KD

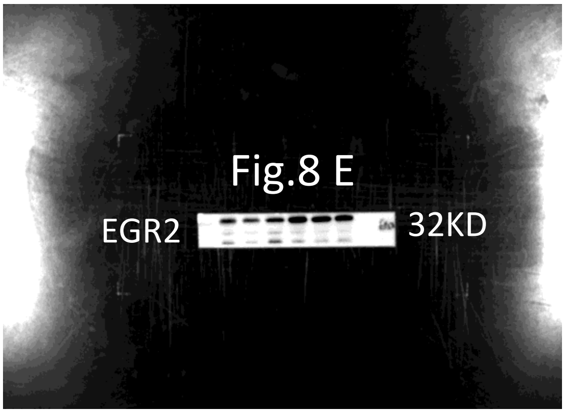
Fig.8 E
EGR2
32KD

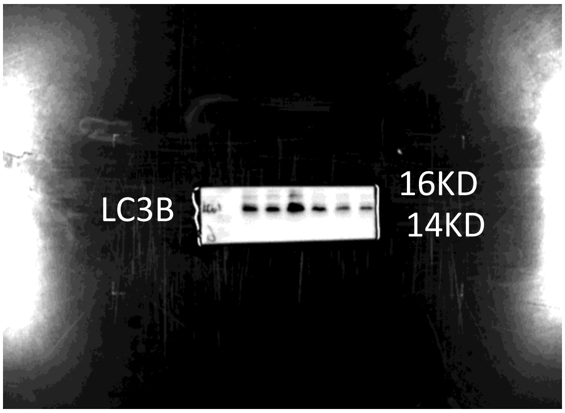
LC3B
16KD
14KD

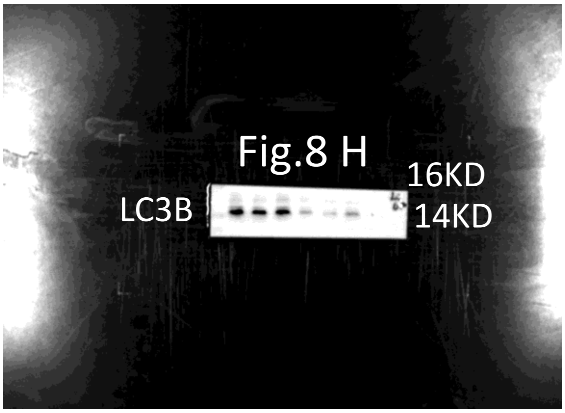
Fig.8 H
LC3B
16KD
14KD

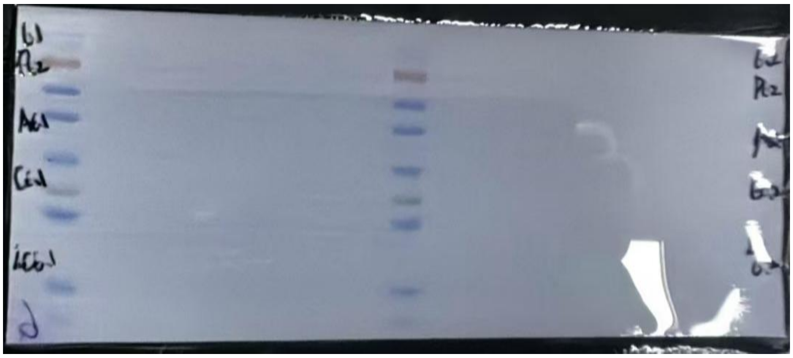

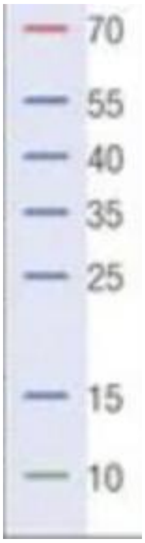
70
55
40
35
25
15
10

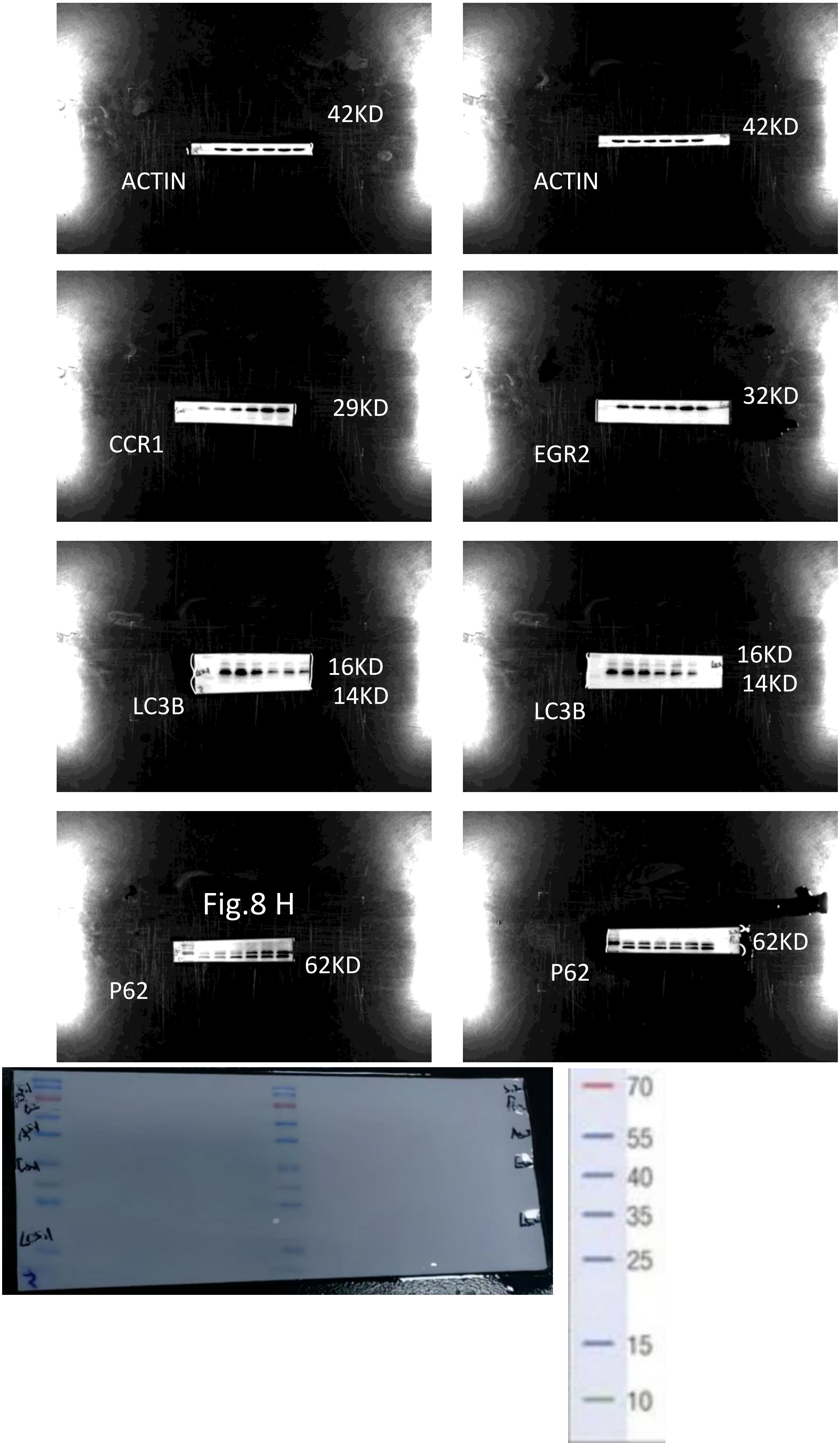
42KD
ACTIN
42KD
ACTIN
29KD
CCR1
32KD
EGR2
16KD
14KD
LC3B
16KD
14KD
LC3B
62KD
P62
62KD
P62
70
55
40
35
25
15
10
Fig.8 H

Actin

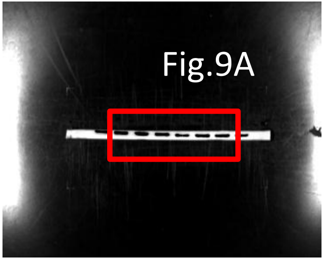

CCR1

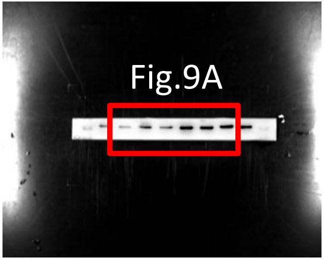

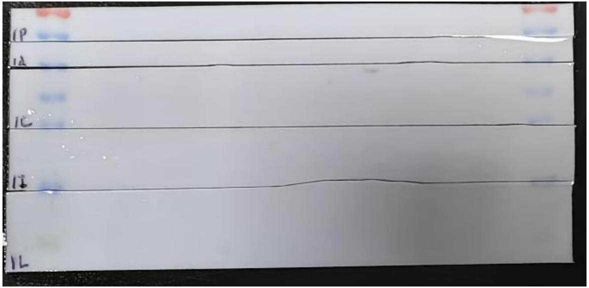

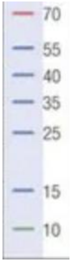

Supplement: Supplementary file 2 — Supplementary Material 2 [file 41598_2026_43737_MOESM2_ESM.pdf]
